# Beyond the Advanced Therapies Skills Training Network: An Instrumental Case Study of Life Sciences Skills Development for Biomedical Science Graduates in Scotland

**DOI:** 10.3389/bjbs.2023.11654

**Published:** 2023-09-04

**Authors:** Claire L. P. Garden

**Affiliations:** School of Applied Sciences, Edinburgh Napier University, Edinburgh, United Kingdom

**Keywords:** skills, training model, evaluation, employability, life science

## Abstract

Biomedical sciences graduates are employed in a variety of different settings and form a significant part of the Life Sciences sector workforce in Scotland. Their degrees should equip them with the skills and knowledge to not only enter the workplace, but be adaptable in an environment that will inevitably change over the course of their careers. Industry and student feedback continue to identify perceived skills gaps, necessitating regular government-backed upskilling initiatives together with industry concerns about graduate readiness. For more than a decade, this Scottish Modern University has worked in partnership with industry and Scottish Government agencies to provide upskilling courses and incorporate relevant skills into the biomedical sciences curriculum, from problem solving and reflection to more applied, practical skills. Using the recent Advanced Therapies Skills Training Network collaboration as an instrumental case study this paper describes current best practice which has significantly impacted teaching and workplace training, ensuring biomedical sciences graduates have the knowledge and skills required for employment within the Life Science sector. Limits to the current life science skills model in Scotland are also identified (availability of placements, ad-hoc and inefficient collaborative structures, incompatible provider strategies) and recommendations made to ensure that biomedical sciences degrees continue to be part of a more sustainable, scalable solution to the skills gap. Recommendations include: better industry acknowledgement of accreditation, and more coherent, authentic and strategic collaboration which should improve skills advice and training, through a supported alliance between Industry and University Life Science Skills Committees and the establishment of regional training Centres of Excellence that would provide a focus for pooled resources and a simulated industry experience.

## Introduction

In Scotland, Life Sciences is a strategically important growth sector worth just under £3.4bn supporting over 38,000 Full Time Equivalent jobs, with the Pharmaceutical sector making up around half [1]. There were 3,500 jobs posted in the sector in 2022, with one third sourced from new graduates [2, 3]. Scotland also has the highest number of higher education students enrolled in Science, Technology, Engineering and Mathematics (STEM) subjects relative to population size, compared the rest of the United Kingdom (UK; [1]. In 2019/20 11 Scottish Higher Education (HE) providers produced 745 biomedical science graduates, rising to 940 in 2021/22 [4]. These providers comprise different types of universities, from those with a traditional research focus through to modern, applied institutions. Biomedical Science degrees act as a gateway to a diverse range of career pathways influenced by the type of accreditation as well as the focus of the provider. Graduates possess a set of core skills that remain in demand in differing contexts and applications that change frequently [5]. For example, critical thinking, innovation and digital skills continue to be in demand in the rapidly changing life science industry, together with more applied knowledge and skills such as clinical chemistry (biomarkers), cytopathology/cellular pathology (microscopy and cell culture), microbiology and immunology, and techniques such as molecular genetics, electrophoresis, flow cytometry, and serial data collection and analysis [5].

The Association of the British Pharmaceutical Industry (ABPI) has worked for nearly 20 years to identify and find solutions to the skills gap they face [6]. This led to the Royal Society of Biology (RSB) accreditation scheme and more recently, a number of commitments to supporting apprenticeships as a solution to skills shortages in the sector [5, 7]. However, there are a number of challenges associated with apprenticeships, including recent decreased applicant demand compared to HE, and decreasing employer demand [7]. Given that there are no *graduate*-level apprenticeships in Life or Biomedical Sciences in Scotland (apprenticeships are available up to SCQF level 7), a significant barrier to sector growth, in Scotland, Biomedical science graduates are already an important part of the life sciences skills landscape [2]. These graduates need to have the appropriate skills to enter the sector to sustain growth. However, undergraduate students in HE are expressing dissatisfaction regarding their skills training [8, 9], and so current best practice is to blend the two approaches by incorporating more industry-appropriate skills into the undergraduate curriculum, supported by industry experience opportunities and accreditation, occasionally enhanced by curricular input from upskilling initiatives.

This paper will present an instrumental case study [10] of the experience of developing and delivering a recent Advanced Therapies Skills Training Network (ATSTN) upskilling course. This case was chosen to illuminate a particular issue, the present limitations of the life sciences skills training model in Scotland. The aim is to showcase current best practices in pedagogical approaches which have significantly impacted teaching and workplace training ensuring biomedical sciences graduates have the knowledge and skills required for employment within the life science sector. The limitations of the current model will be explored, giving recommendations for sustainable future development including an examination of the three available accreditation frameworks in the UK from the point of view of life sciences skills to support this aim.

## The Current Life Science Skills Model in Scotland

There are a mixture of providers of Life Science Skills training in Scotland, including colleges, universities and industry, reflected in the make-up of the recent ATSTN consortium [11]. The SFC funds qualifications in relevant subjects at colleges (who also support apprenticeship), together with university degrees for Scottish students, and have recently instigated a coherence and sustainability review of tertiary education and research with the aim of driving more strategic collaboration amongst providers [12]. Each university is committed to an outcome agreement as a result of this funding, and performance is measured through UK-wide graduate outcomes and student satisfaction surveys (there is no Teaching Excellence Framework in Scotland as Education is a devolved matter [13]). In addition, universities also participate in the Research Excellence Framework as a measure of their research activity. Although all universities undertake teaching, the balance of research-led and applied teaching activity differs according to the strategic priorities of each institution and so due to differing priorities some are better placed to contribute to sector growth through addressing skills shortages than others. Indeed, graduates from any biology or biomedical science(s) degree are able to enter a variety of professions [5], and a few Scottish universities offer an industry-focussed undergraduate curriculum, whereas others specialise in training biomedical scientists to seek registration with the Health and Care Professions Council (HCPC) to work under the protected title of Biomedical Scientist, or prepare graduates to enter the research route. Regardless of the supply of new college and university graduates, employers continue to rely on on-boarding and in-work skills training, and regularly report issues to do with graduate readiness [2]. These are addressed through upskilling initiatives sporadically funded by various government agencies and delivered through industry-academia partnerships.

**FIGURE 1:**
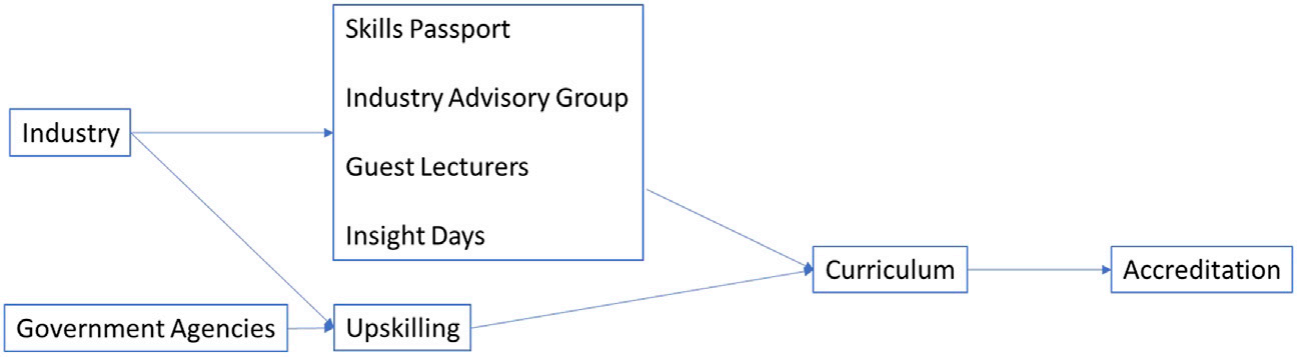
Current best practice for incorporating life sciences skills into the Scottish undergraduate curriculum as developed at a Scottish Modern University. The model describes institution-level partnerships between industry, government agencies and the university which are used to support the development and implementation of a skills passport, industry advisory group, guest lectures, insight days and upskilling which influence the curriculum as evidenced by accreditation.

For more than a decade this Scottish Modern University has worked with partners in industry to provide solutions to these challenges by incorporating life sciences skills development into the undergraduate biomedical sciences degree. Industry-led or informed teaching activities rely on significant partnership working between industry, either directly or via various associations, and universities because the academic staff base is predominantly trained via the research route with limited experience of working in industry. To meet this need, we established an Employer Liaison group a decade ago, now called the Industry Advisory group, to inform our life science curricula, and invested in industry-experienced academic colleagues. In 2015 £2.7m Scottish Funding Council (SFC) -funded Graduate Employability Project supported the development and implementation of a skills passport [8, 9]. Shortly after, our Biological Sciences undergraduate suite of degrees (including biomedical sciences) were among the first in Scotland to achieve RSB accreditation in recognition of this approach, with the accreditation being renewed recently. Since then we have been regularly awarded small amounts of funding (totalling £90,000) to provide collaborative upskilling courses with industry such as laboratory skills and quality assurance. We incorporate aspects of these into our taught degree curricula, including guest lectures and industry insight days, ensuring that these courses have an impact beyond the participants, influencing the education of hundreds of students (Figure 1). These courses are funded by, and delivered on behalf of government agencies, most recently we were the only Scottish University partner in the Cell and Gene Therapy Catapult -funded ATSTN consortium.

Most partnership working currently takes place at an institutional level, although a national industry guidance board to assist outreach has been suggested to assist with cooperation [2]. There is an active Industry Leadership Group and Scottish Cross Party Life Sciences Group in Scotland with some university representation, the Scottish Universities Life Sciences Alliance (SULSA) being the main conduit to academia for the purposes of skills collaboration [2, 14–16].

## The Advanced Therapies Skills Training Network Case

The ATSTN in Scotland brought together industry with SFC funded entities including one Scottish Modern University as the sole university partner [11]. The £664k consortium was funded by Innovate UK and the Department for Business, Energy and Industrial Strategy via the Cell and Gene Therapy Catapult to provide upskilling to meet the needs of the accelerated growth of this part of the Life Science sector [2]. UK employee headcount in cell and gene therapy and/or vaccine manufacturing more than doubled in the period from 2019 to 2021, and is forecast to double again by 2026 [2].

Our 5 days intensive courses were designed in partnership with an industry partner, and incorporated over a decade of experience delivering upskilling courses and industry-relevant skills within the undergraduate degree in order to meet the aims of the project (Table 2). We quickly identified basic laboratory skills (including pipetting), cell culture, and an awareness of medicines regulation (including Good Laboratory Practice, GLP), governance and standards as the core curriculum for the courses, as these skills gaps are regularly reported by industry and were already being addressed to some extent in our undergraduate curriculum [2, 9]. We added some knowledge-based content on advanced therapies to provide context and sought to provide as much industry-authentic hands-on practical experience as possible. The courses were delivered 9am–5pm to encourage “at-work” professional behaviours and attitudes and were accompanied by a set of workbooks and Standard Operating Procedures. Throughout the course, opportunities to develop numeracy, network and to undertake personal development/careers planning were embedded. Each week-long course incorporated 1 day of lab experience in industry undertaking molecular biology and immunoassay, gaining a practical understanding of GLP. Three days of practical experience at the university included cell culture, core laboratory skills, immunoassay and microscopy. The course culminated in 1 day of medicines regulation, governance and standards theory delivered by industry partners and the award of a certificate of completion.

The project was jointly developed before the impact of the COVID-19 was felt across the Higher Education and Life Sciences sectors. The pandemic necessarily constrained its delivery, delaying the first course until January 2022. Recruitment to the first course was limited to current students at this institution due to health and safety requirements at time. Participant numbers were limited to 24 (four groups of six participants) per course to ensure that labs and teaching spaces were COVID-19 compliant and the first cohort comprised current postgraduate or final year undergraduate students at the university because of the difficulty in releasing employed participants during a high demand period in 2022, and the buoyant employment market for recent graduates. In total, three courses were delivered in 2022 to 68 students from eleven universities and a local college, with recruitment supported by SULSA. Course tutors comprised fourteen members of staff working with our industry partner and eight academics who had either previously worked in industry or had recent experience in collaborative industry-relevant curriculum development. Tutor biographies were included in the participant handbook, and participants benefited from networking with tutors, hearing a number of different career paths involving the Life Sciences Industry. Participants were encouraged to continue to network after the course via LinkedIn.

## Course Evaluation

The short courses achieved positive results in an evaluation administered via Mentimeter on the final day of the course (Table 1), with participants reporting an increased interest in working in industry and improved confidence in their practical skills as a result of the course. This was especially poignant given the context of delivering these courses during the COVID pandemic. The combination of laboratory skills training and industry partnership delivery and insights appeared to work well for the courses with “lab work,” “people/networking” and “industry insights” ranking as the top three items participants enjoyed most about the week. Indeed, the biggest learning point from the course was “working in a regulated lab/industry standards,” “lab skills/confidence” and “careers insights.”

**TABLE 1:** Course evaluation including modified Careers Readiness Survey questions.

Question	Question type	Number of responses
*What is your biggest learning point from the course?*	Open ended	37
*What action will you take as a result of the course?*	Open ended	31
At the end of the course I feel	Sliding scale: strongly disagree- strongly agree	37
More Confident in the lab		
I understand more about how the Life Sciences Industry works		
I understand what roles might suit me in the Life Sciences Industry		
More able to apply for jobs in the Life Sciences Industry		
I have a clear plan of what to do next to achieve my career goals		
*Tell us about your career plans*	Sliding scale: strongly disagree- strongly agree	56
I have not decided what career I want to follow after my degree		
I have a career in mind but need to research it		
I have a career in mind but need some experience		
I feel ready to apply for a job		
I already have a job or further study ready		
I am interested in working in the Life Sciences Industry		

Participants answered all questions positively from a modified careers readiness survey included in the evaluation [17], indicating a positive impact of the course on the participant’s careers readiness. Many participants planned to continue their journey towards a career in the Life Sciences industry after the course through further research, training or job applications. This initiative met the following needs as described in the 2021 UK Cell and Gene Therapy Skills Demand Survey Report (Table 2, [2]).

**TABLE 2:** The ENU ATSTN Course met needs as described in the 2021 UK Cell and Gene Therapy Skills Demand Survey Report [2].

Need [2]	Precursor at ENU	Realisation	Legacy
Short training courses; Training programmes to “seed the market” with skills	Previous upskilling courses in laboratory skills and Quality Assurance and Regulatory Affairs; industry-relevant curriculum	3 × 1 week ATSTN courses	Incorporate content and learnings into curricula
Transparent schemes to get people into industry	industry-relevant curriculum, RSB accreditation (undergraduate)	Included 1x industry lab day and 1x industry theory day Certificate provided	Incorporate content and learnings into curricula
Increased number of free courses on Online Training Platform	SFC funded undergraduate degrees	Face to face course free at point of delivery to participants	Incorporate content and learnings into undergraduate (SFC funded) curricula
Identify and recognise transferable skills	Skills passport [9], RSB accreditation	Partnership approach to development, certificate	Continued RSB accreditation, dissemination of course content and outcomes, contribute to sector discussion on Graduate Readiness
Attracting people with potential	SFC funded undergraduate degrees	Recruitment of 65 students from 10 Scottish Universities	Incorporate content and learnings into undergraduate (SFC funded) curricula
Attracting people to work in Manufacturing and Quality roles and in Good Manufacturing Practice environments	Legacy content from Quality Assurance and Regulatory Affairs upskilling, relevant guest lecturer workshop in existing 4th year module	Inclusion of Quality content and case studies and networking with relevant role holders in course	Continuation of delivery of theory day after end of ATSTN course in addition to previous guest lecturer content
Creating an industry guidance board to assist outreach; Need to work as a community to share talent	Existing institutional Industry Advisory Group	SULSA Liaison for project	Institutional partnerships with industry; SULSA Skills committee for member university collaborations
Industry experience or relevant transferable skills	Industry-relevant curriculum, Skills passport [4], RSB accreditation	Included 1x industry lab day and 1x industry theory day	Institutional partnerships with industry; SULSA Skills committee for member university collaborations

Our students already benefit from the Skills Passport [9], which includes the introduction of GLP type activities early in their course and an emphasis on laboratory skills. However, the ATSTN courses provided valuable lab experience, having had reduced face-to face lab teaching during the pandemic (lab-based honours projects continued as usual), and reached participants beyond the university, leading to employment for some. As a legacy to the project, we are continuing to offer the industry lab and theory days to our students, and plan to add a clean room experience and networking opportunity with another industrial partner.

The strength of project was the continuation and deepening of collaborative partnerships between industry and academia in the design and delivery of the courses. This led to the establishment of the SULSA Skills Committee which continues to support collaboration on the skills agenda between member universities. However, despite the option to use pooled resource to work across companies being a theme across multiple respondents to the 2021 UK Cell and Gene Therapy Skills Demand Survey Report [2], which led to the ATSTN project, there are no plans to continue to fund collaboration between Scottish industry and academic partners in a sustained and coordinated way. This will severely limit the potential legacy of the project.

## Discussion

The ATSTN courses we led were a great success for participants, who received both industry experience and relevant skills training in line with the project aims (Table 2). Furthermore, the collaborating institutions also continue to benefit from the legacy of sustained curriculum development and partnership delivery (university partner) and employment of successful candidates (industry partners). One project aim was to establish an industry quality trademark to know “good” programmes. Although not established during the project, accreditation frameworks for degrees that incorporate aspects of this content already exist (Table 3). The requirement for grants to support social mobility through Equality, Diversion & Inclusion was also beyond the scope of our part of the project, however, Scottish universities continue to meet this aim via their SFC outcome agreements.

**TABLE 3:** Life science industry-relevant skills accreditation for biomedical science or pharmaceutical sciences degrees.

Industry requirement	Requirement source	Accreditation framework (s)	Barriers and limitations	Solution
Core subject knowledge, e.g., physiology, cell and molecular biology, pharmacology, etc.	QAA Subject Benchmark [18, 23]	IBMS [20]; RSB [19]; APS [21]	N/A Universities are experienced in knowledge transmission	
Core professional skills, e.g., reflection, numeracy, critical, analytical, research, group work, etc.	QAA Subject Benchmark [5, 18, 23]	IBMS [20]; RSB [19]; APS [21]	Student skills and knowledge on entry	Integrated academic Skills support
Entrepreneurship	QAA Subject Benchmark [1, 16, 21](1)	RSB: an understanding of the interdisciplinary nature of enterprise [19]	Student skills and knowledge on entry	Integrated support from University Centres for Entrepreneurship
Digital Skills	[3, 23]		Student skills and knowledge on entry	Integrated academic Skills support
Laboratory skills	[23]	IBMS [20]; RSB [19]; APS [21]	Limited teaching lab space, growing student numbers. Access to applied knowledge, spaces and equipment	Blended approach using online simulations, VR and video: guest lecturers, pooled resources at Centre of Excellence for Skills and Training, Simulated Placements
Safe Working Practices, e.g., risk assessment	QAA Subject Benchmark [18]	IBMS [20]; APS [21]	N/A Universities are experienced in safe laboratory practice	
Applied Knowledge: Medicines Regulation, e.g., GxP; Governance and Standards	[3]	IBMS mentions GLP, compliance, governance and audit [20]; RSB mentions GLP and regulatory issues [19]; APS [21]	Academic skills and knowledge	Guest lecturers, central coordinated Centre of Excellence for Skills and Training, Simulated Placements
Placement/Industry Experience	QAA Subject Benchmark [18]	IBMS [20]; RSB mentions contextualised learning [19]; APS [21]	Industry capacity [7]	Industry Advisory Groups, Simulated Placements

### Accreditation

There remains an ongoing requirement to embed skills in undergraduate degrees to continue to improve graduate readiness, expanding the work we already undertake beyond our institution. To an extent, accreditation serves this purpose by setting skills and knowledge requirements for accredited degrees. We chose RSB accreditation because of the breadth of our provision and the explicit industry focus of the accreditation framework, which matched our own [5].

The Quality Assurance Agency (QAA) has published benchmark statements necessary for undergraduate Biomedical Science(s) programmes [18] and there exist three accreditation schemes in the UK for courses relevant to the life science industry. Most universities choose one or more aligned to their strategic aims and the needs of the degree. Each is different because of the professions they serve: the Royal Society of Biology (RSB) has the broadest and most flexible framework which aims to, among other things: “enhance competitiveness for students in a global jobs market; provide industry with an assurance of the level of employability skills and subject relevant bioscience skills provided by a programme; maintain and improve the UK’s position as a premier location to develop the life scientists of the future” [19]. By contrast, the Institute of Biomedical Science (IBMS) accreditation aims to “meet the Health and Care Professions Council (HCPC) standards of proficiency for biomedical scientists” by ensuring that a degree course covers the academic components of these standards, with a further certificate of competence required to demonstrate an individual’s full adherence with them [20]. The new Academy of Pharmaceutical Sciences (APS) Curriculum framework has been established to promote good practice in the training and development of pharmaceutical scientists and supports two of the APS strategic themes: “establishing and promoting the reputation of pharmaceutical sciences and scientists and promoting careers” [21]. However, this scheme seeks to accredit *pharmaceutical* sciences degrees with a minimum of 65% pharmaceutical content, which may be limiting for some university offers.

The core industry requirements (synthesised from [22–24] are mapped against each accreditation framework below (Table 3). This shows that the three schemes, although they each have a different emphasis, do fulfil most of these requirements, except for Digital Literacy, which was not mentioned explicitly in any of them. Unfortunately, despite the RSB scheme running for over 8 years now, and IBMS accreditation reaching back decades, industry still identified a requirement for a “quality trademark to know good programmes” offering core skills training in a recent survey [2]. Therefore, accreditation alone is unlikely to meet this need without better industry awareness of the underpinning frameworks and buy in for partnership delivery. We implement an enhanced accredited curriculum via a dedicated Professional Practice module and the Skills Passport [9]. In order to future-proof student learning in a rapidly changing landscape we prioritised student-focussed reflection, core skills such as problem solving and laboratory skills, and flexibility over competency-based assessment [5, 8]. However, there is an ongoing requirement for collaboration to continue to meet employer needs and core curriculum changes which we manage through our Industry Advisory Group and guest lecturers. A more sustainable solution with impact beyond this institution may be found by supplementing accreditation through future partnership work to develop a competency framework linked to core skills, knowledge and behaviours, via a regional coordinated Skills and Training Centres of Excellence, delivered through simulated placements assessed by industry.

### Industry Experience and Relevant Skills

A 1 week upskilling course cannot meet all the needs of students and demands of a growing industry [5], serving only as an introduction to relevant specialist skills and experience for a limited number of participants. The 1 day industry lab experience was prized by all involved, but was no substitute for a longer, more immersive industry placement that would have consolidated and strengthened industry-relevant skills and behaviours, something that is desirable but generally lacking in degrees [7]. Unfortunately, life science placements and internships are expensive and do not meet demand, with 697 provided in total in 2022 across the UK, almost double 2009 levels [25], fewer than the number of Biomedical Sciences graduates Scotland alone produce in a year. These workplace experiences are also vulnerable to economic impacts, affecting reliability and sustainability: “When you build a skills system that relies heavily on employers being able to offer jobs or placements, then what happens at the point where employer capability to do that reduces, say in a recession, when you have more young people wanting to take those opportunities?” [7].

Given that industry placements and upskilling initiatives reach limited numbers of individuals, an alternative must be sought. Indeed, the Quality Assurance Agency states the following in its Benchmark Statement: “2.17 Courses should work with relevant stakeholders to incorporate work-based or work-like learning where possible. Enhancing student employability is a fundamental outcome for Biomedical Science and/or Biomedical Sciences courses. Therefore, engagement with the relevant employment sectors should be extensive. The courses should have a clear strategy for students to have the opportunity to develop employment-focused skills and engage with employers. Students may engage with employers through paid and/or unpaid placements of various durations during which students will be fully immersed in the workplace and experience the day-to-day routine of employment” [18]. A centralised work-like training offer may be one solution to this persistent issue.

### Centralised Life Sciences Skills and Training

Life sciences research and innovation has received sustained funding and contributes to the success of the sector in Scotland, the subject of a number of recent reports [1, 3, 24, 26]. Close links and partnership working between the private sector, NHS Scotland and University research have been central to the strength and growth of the sector [1], and there is a similar, unmet, requirement for coordinated partnerships and activities to grow skills training that would reach more people to meet demand in Scotland [24].

The ATSTN initiative went some way to deliver its upskilling aims, although growing demand will not be met without sustainably scaling up life sciences skills training in Scotland [2]. Indeed, not much has changed in over a decade with regards to the training approach, a mixture of upskilling and degree content [22]. Scaling up training will require radically changing the model away from limited institution-level partnerships towards more centralised, strategic pooling amongst key stakeholders. This would allow many universities to work together with colleges, industry and government agencies to incorporate industry-informed skills training and experiences into their degrees whilst contributing their research, learning and teaching expertise. Given the complicated stakeholder landscape in Scotland, sustained and strategic collaboration between colleges and universities, industry and government agencies to inform and develop curriculum is difficult. Indeed, the option to use pooled resource to work across companies was a theme across multiple respondents to the 2021 UK Cell and Gene Therapy Skills Demand Survey [2]. These partnerships can be difficult for individual universities to resource, with industry struggling to meet demand from multiple sources. Although a national skills committee for industry has been established for some time, its university equivalent has only recently been put in place, supported by SULSA. These now require support for strategic collaboration to unleash the potential for more efficient, transformative partnership working. Furthermore, mirroring the National Horizons Centre in England, regional Life Science Skills and Training Centres of Excellence in Scotland would be required in order to maintain competitiveness north of the border. This would also support collaboration between the various SFC-funded providers, including universities, meeting a recent SFC strategic requirement for sector collaboration and consolidation [12].

This requirement for supported collaboration is doubly important because life sciences is different to the professions such as law, teaching, nursing and medicine, and the arts where teaching and practice are integrated. Most academics responsible for developing and teaching life science courses have a research or clinical background and lack recent industry skills and experience required to design and deliver applied/industry-relevant content. Therefore, if teaching is to be relevant to cutting edge applications with up to date, context-dependent materials, sustained partnership working with industry is required. Collaboration via regional Life Science Skills and Training Centres of Excellence offering full access to industry experience and relevant skills training, together with the implementation of a relevant competency-based curriculum/accreditation framework for degrees would be a fruitful area for future development that would meet market demand for skills and support growth of the sector.

**FIGURE 2:**
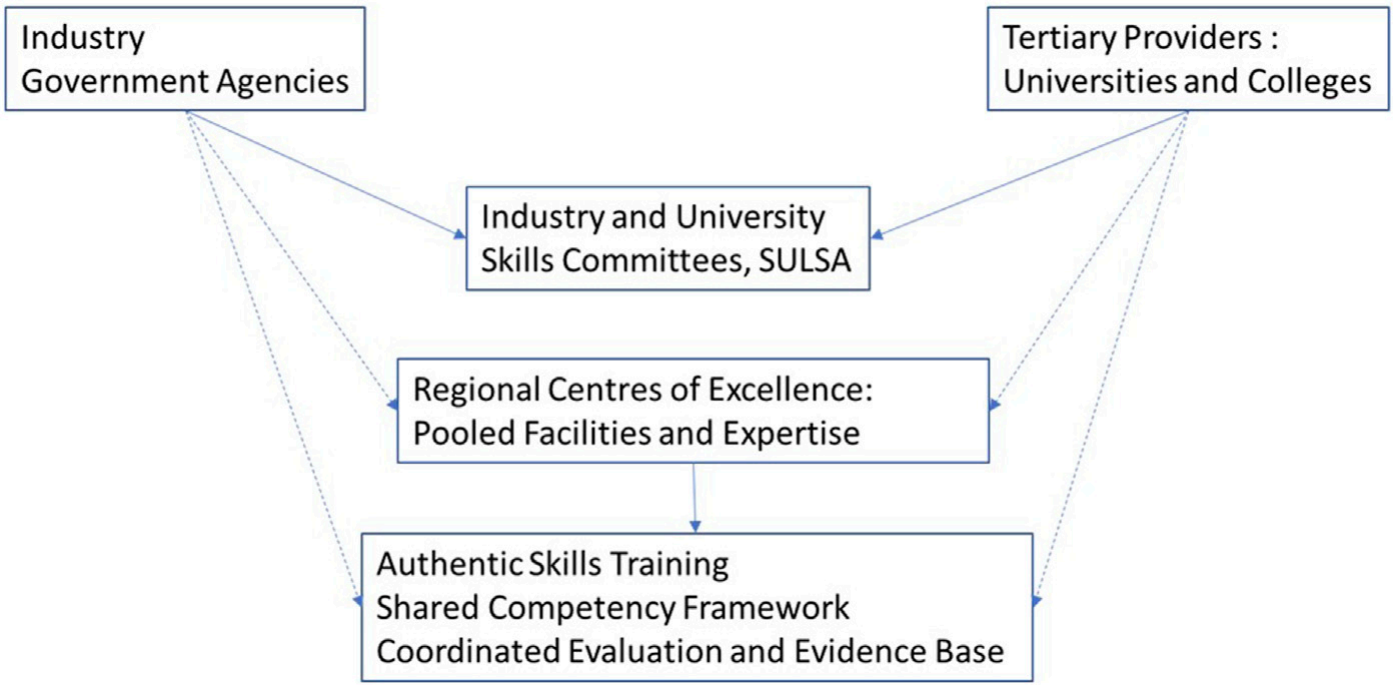
Regional Centres of Excellence would bring together stakeholders in Scotland in a strategic, efficient and sustainable way to properly support partnerships and realise the potential to meet workforce requirements. Skills committees (solid arrows) are now in place, with SULSA playing a strategic role in bringing together stakeholders. In the future, Regional Centres of Excellence could act as a focal point for pooling facilities and expertise, leading to a more impactful skills offer through shared training, curriculum and evaluation (dotted arrows).

Regional Centres of Excellence would bring together stakeholders in Scotland in a strategic, efficient and sustainable way to properly support partnerships and realise the potential to meet workforce requirements. This would also continue to improve harmonisation and access to meet skills demand, and the need to work as a community to share talent [24]. Regional Centres of Excellence could provide an environment where placements could take place in an authentic space co-designed with industry partners, using skills training competencies and equipment developed in partnership, assessed by industry trainers and acknowledged in university degree structures (Figure 2). Innovations such as blended learning and virtual reality could be incorporated to maximise the benefit of the practical training and experience, contextualised by industry-relevant scenarios. Cell and gene therapy is only one application for life science skills training provided by university degrees. There is high export-growth potential in: Precision medicine; Regenerative medicine and tissue repair; Preclinical drug development; Clinical trials and preclinical services; Biopharmaceutical safety testing; Specialist and high value manufacturing and Regulatory support applications [1], so the option to vary the context should be borne in mind as the context for core skills training with some flexibility around specialist laboratory skills is likely to continue to change. This “simulated placement experience” is a recommended area for future work.

## Recommendations and Future Directions

The ATSTN project serves as an instrumental case study demonstrating the mutual benefits to biomedical science graduates and the Life Science Industry of continued collaboration between academia and industry to develop and embed relevant skills training into their curriculum. To date there has been no initiative to scale up this type of life sciences skills provision in a sustainable way to support the strategic growth of the sector in Scotland. This is due to a number of barriers, as outlined above. A summary of recommendations that would mitigate these barriers and support growth in industry-relevant life science skills training is found below:

1. Support and strengthen collaboration between national Industry and University Skills Committees.
2. Establish regional Life Science Skills and Training Centres of Excellence to coordinate academia-industry collaborative life science skills provision and host an authentic industry designed environment.
3. Develop, implement and evaluate an authentic Simulated Placement offer based on industry-relevant competencies and behaviours assessed by industry partners to be delivered at the central space.
4. Encourage industry engagement with and recognition of accreditation as a means to identify high quality courses.

These recommendations require sustained investment, and would be designed to be flexible and applicable to the emerging specialties in the sector. This investment would also incentivise universities to take part where competing strategic priorities currently temper engagement. Partnership working between academia and industry will remain a high priority with universities contributing research and teaching expertise for curriculum/model development to ensure effective and evidence-based interventions, whilst industry contribute context-dependent expertise and materials. Building in evaluation of outcomes would ensure that the value of any intervention is measured and disseminated for long term impact.

## Limitations

The paper focuses on a single Scottish University and upskilling course as an instrumental case study [10]. By definition, this is a case chosen to illuminate a particular issue, here the issue is the present limitations of the life sciences skills training model in Scotland. It is appreciated that other Scottish and UK providers are also working in this space, and they will likely recognise the challenges outlined.

## Data Availability

The original contributions presented in the study are included in the article/supplementary material, further inquiries can be directed to the corresponding author.
